# Effects of dietary Lactobacillus rhamnosus GG supplementation on the production performance, egg quality, eggshell ultrastructure, and lipid metabolism of late-phase laying hens

**DOI:** 10.1186/s12917-023-03719-9

**Published:** 2023-09-08

**Authors:** Liming Liu, Guoqing Zhang, Ge Qu, Bin Liu, Xiufeng Zhang, Gaoqian Li, Ningyi Jin, Chang Li, Jieying Bai, Cuiqing Zhao

**Affiliations:** 1 College of Animal Science and Technology, Jilin Agricultural Science and Technology University, Jilin, 132101 Jilin China; 2 Jilin Genet-Med Biotechnology Co., Ltd, Changchun, 130122 Jilin China; 3 Research Unit of Key Technologies for the Prevention and Control of Virus Zoonoses, Changchun Veterinary Research Institute, Chinese Academy of Medical Sciences, Chinese Academy of Agricultural Sciences, Changchun, 130122 Jilin China; 4 College of Future Technology, Peking University, Beijing, 100871 China

**Keywords:** *Lactobacillus rhamnosus* GG, Laying hens, Production performance, Eggshell quality, Lipid metabolism, Eggshell ultrastructure

## Abstract

**Background:**

Toward the late phase of laying, the production performance of laying hens decreases, egg quality deteriorates, lipid metabolism weakens, and hepatic lipid accumulation is exacerbated. Probiotics as an alternative to antimicrobials have been employed in poultry-related industries. *Lactobacillus rhamnosus* GG (LGG) is currently the most researched and clinically validated probiotic, showing promising effects in multiple application areas. However, few studies have been conducted on livestock (including poultry) production.

**Results:**

Compared with the CON group, the feed conversion ratio (P < 0.01) declined significantly in the LGG group. Eggshell strength (P < 0.001) and eggshell thickness (P < 0.001) were significantly increased by supplementation with LGG in the diet. The height (P < 0.001) and proportion (P < 0.05) of the effective layer and the mammillary knob density (P < 0.01) in the eggshell ultrastructure of the LGG group increased significantly, while the mammillary layer (P < 0.05) and knob width (P < 0.01) decreased significantly. The LGG-treated hens had significantly lower serum concentrations of low-density lipoprotein (P < 0.05), free fatty acids (P < 0.01), and liver triglyceride (P < 0.05) levels than those in the CON group.

**Conclusions:**

LGG supplementation significantly decreases the feed conversion ratio, improves eggshell quality by altering the ultrastructure, and improves lipid metabolism in the late laying period.

## Background

Eggs, which contain a variety of fatty acids, vitamins, and minerals required for human health, are an important and inexpensive source of high-quality protein, and the demand for this popular and nutritious food is increasing. The industrialization of the poultry industry has resulted in longer feeding periods. Consequently, the metabolic capacity and physiological function of laying hens tend to gradually decline after their peak laying period, which requires a high metabolic output. As a result, the production performance and egg quality of laying hens rapidly declines in the late laying period [1], which has serious financial impacts on farmers. Therefore, there is a need to improve egg production and quality in the late laying period to extend the production cycle and raise the breeding efficiency of laying hens [2, 3].

Probiotics are living microorganisms that are administered in feed to confer health benefits. They have been increasingly used in animal breeding following the prohibition of antibiotics in feed (European Union since 2006 and in China since 2020). At present, a variety of probiotics have been employed in poultry-related industries. Previous reports have demonstrated that probiotics can reduce ammonia emissions in chickens, inhibit pathogen colonization, and maintain the balance of the gastrointestinal microbiome [4, 5]. It has also been reported that dietary probiotics can enhance feed intake, energy utilization, growth performance, egg quality, and immunity while maintaining the intestinal health of poultry [6–10]. In addition, probiotics are able to reduce fat deposition in the liver and to improve indicators of lipid metabolism in the serum of laying hens [11–14].

*Lactobacillus rhamnosus* GG (LGG) is currently the most researched and clinically validated probiotic around the world and is also the most functional probiotic. Clinical studies have shown that LGG exhibits beneficial effects such as balancing intestinal flora [15, 16], improving intestinal health [17], enhancing the intestinal mucosal barrier [18, 19], preventing and treating diarrhea [20], improving immunity [21], preventing and promoting allergic recovery [22], and regulating glucose and lipid metabolism [23, 24]. It is worth noting that in our previous studies, LGG significantly reduced hepatic lipid accumulation and liver injury by regulating intestinal barrier function and improving lipid metabolism [25–27]. However, there have been no reports of the use of LGG in poultry production. Especially in the late stage of laying, the production performance of laying hens decreases, egg quality deteriorates, lipid metabolism weakens, and hepatic lipid accumulation is exacerbated. Hence, the current study aimed to determine the potential effects of LGG on the production performance, egg quality, eggshell ultrastructure, and lipid metabolism of laying hens in the late laying period.

## Results

### Production performance

All laying hens were healthy throughout the entire experimental period. No obvious differences in the total egg weight and daily laying rate were found between the CON and LGG groups in all three phases (Table 1). Compared to the CON group, the LGG group fed the diet containing LGG had lower feed conversion rates at 31–45 days (*P* < 0.01). Nonetheless, no difference was found in the other phases (Table 1).

**Table 1:** Effects of dietary supplementation of LGG on the production performance of laying hens

Item	Phase of Experiment	CON^1^	LGG^2^	Pooled SEM^3^	*P*-value
Total egg weight (g)	1–15 days	1317	1423	34.6	0.100
	16–30 days	1378	1461	37.3	0.270
	31–45 days	1371	1488	31.3	0.0600
Feed conversion ratio (Kg feed/ Kg egg)	1–15 days	2.20	2.21	0.0500	0.990
	16–30 days	2.21	2.17	0.0500	0.900
	31–45 days	2.30	2.10	0.0400	0.0100
Daily laying rate (%)	1–15 days	68.9	74.4	1.82	0.150
	16–30 days	74.0	75.6	1.95	0.930
	31–45 days	73.6	76.2	2.10	0.720

^1^CON: basal diet

^2^LGG: basal diet supplemented with 8 × 10^10^ CFU/Kg LGG

^3^Data are expressed as the means and pooled standard error of the mean (Pooled SEM)

### Egg quality

Table 2 shows the egg quality results. There were no differences in the egg shape index, Haugh unit, or yolk color between the LGG and CON groups. Eggshell thickness (*P* < 0.01) and eggshell strength (*P* < 0.001) in the LGG group were markedly higher than those in the CON group.

**Table 2:** The effect of dietary LGG supplementation on egg quality traits

Item	CON^1^	LGG^2^	Pooled SEM^3^	*P*-value
Egg shape index	1.43	1.42	0.0100	0.670
Eggshell thickness (mm)	0.380	0.400	0.0100	0.0060
Eggshell strength (N)	30.8	34.1	0.546	0.0003
Haugh units	61.2	63.6	0.915	0.103
Yolk color	10.4	10.8	0.159	0.130

^1^CON: basal diet.

^2^LGG: basal diet supplemented with 8 × 10^10^ CFU/Kg LGG.

^3^Data are expressed as the means and pooled standard error of the mean (Pooled SEM).

### Eggshell ultrastructure

**Fig. 1:**
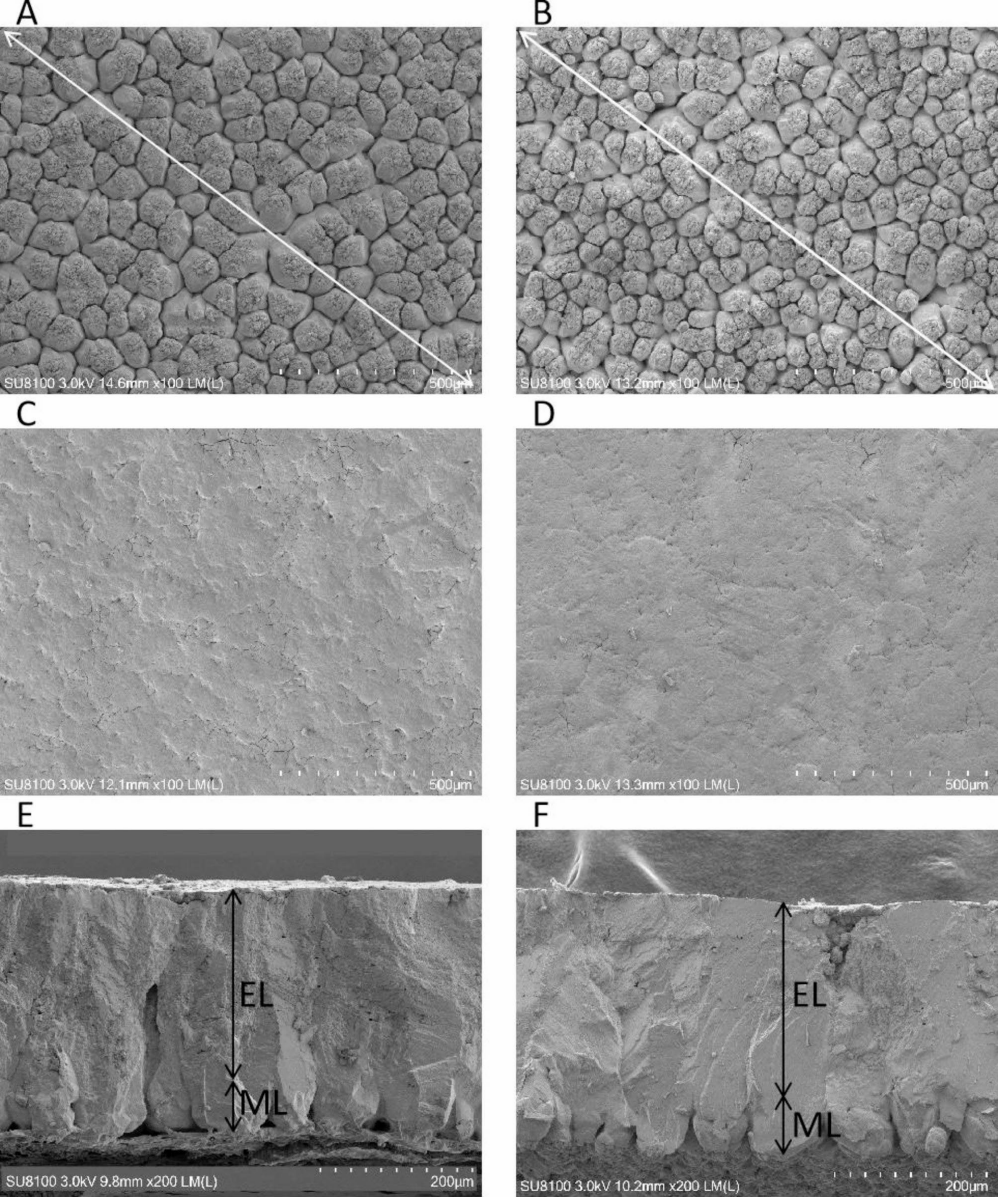
Scanning electron microscopy of the eggshell. The inner surface of the eggshells fed CON (**A**) or LGG (**B**), Scale bar: 500 μm. The outer surface of the eggshells fed CON (**C**) or LGG (**D**), Scale bar: 500 μm. Cross-section of the eggshells fed CON (**E**) or LGG (**F**), Scale bar: 200 μm. CON: basal diet; LGG: basal diet supplemented with 8 × 10^10^ CFU/Kg LGG; EL = Effective Layer, ML = Mammillary Layer

To investigate the effect of LGG on eggshell ultrastructure, eggshell samples from each group were examined by scanning electron microscopy (Fig. 1) and analyzed with ImageJ software (Table 3). The average diameter of the mammillary knob was smaller in the LGG group than in the CON group, indicating that the mammillary knob density was higher in the LGG group than in the CON group (Fig. 1A, B). Moreover, the presence of cracks on the outer surface was significantly reduced (Fig. 1C, D). In addition, the effective thickness of eggshells was significantly increased in the LGG group (Fig. 1E, F). The ultrastructure of the eggshells was observed by electron microscopy. Compared to those of the CON group, the diameter (*P* < 0.001) and the average size of the mammillary knob (*P* < 0.01) of eggshells were significantly reduced in the LGG group. In addition, the effective layer thickness (*P* < 0.0001), effective layer ratio (*P* < 0.05), and total eggshell thickness (*P* < 0.0001) were significantly elevated, and the mammillary layer ratio (*P* < 0.05) was significantly decreased.

**Table 3:** The effect of dietary LGG supplementation on eggshell ultrastructure

Item	CON^1^	LGG^2^	Pooled SEM^3^	*P*-value
Mammillary knobs density (mm^2^)	160	246	8.98	0.0003
The average diameter of the mammillary knob (µm)	77.2	63.4	2.63	0.0047
Effective Layer (µm)	252	295	4.78	< 0.0001
Mammillary Layer (µm)	72.3	70.4	2.88	0.670
Total thickness (µm)	324	366	3.35	< 0.0001
Effective Layer (%)	77.7	80.7	0.910	0.0400
Mammillary Layer (%)	22.3	19.3	0.900	0.0400

^1^CON: basal diet

^2^LGG: basal diet supplemented with 8 × 10^10^ CFU/Kg LGG

^3^Data are expressed as the means and pooled standard error of the mean (Pooled SEM)

### Lipid metabolism

**Fig. 2:**
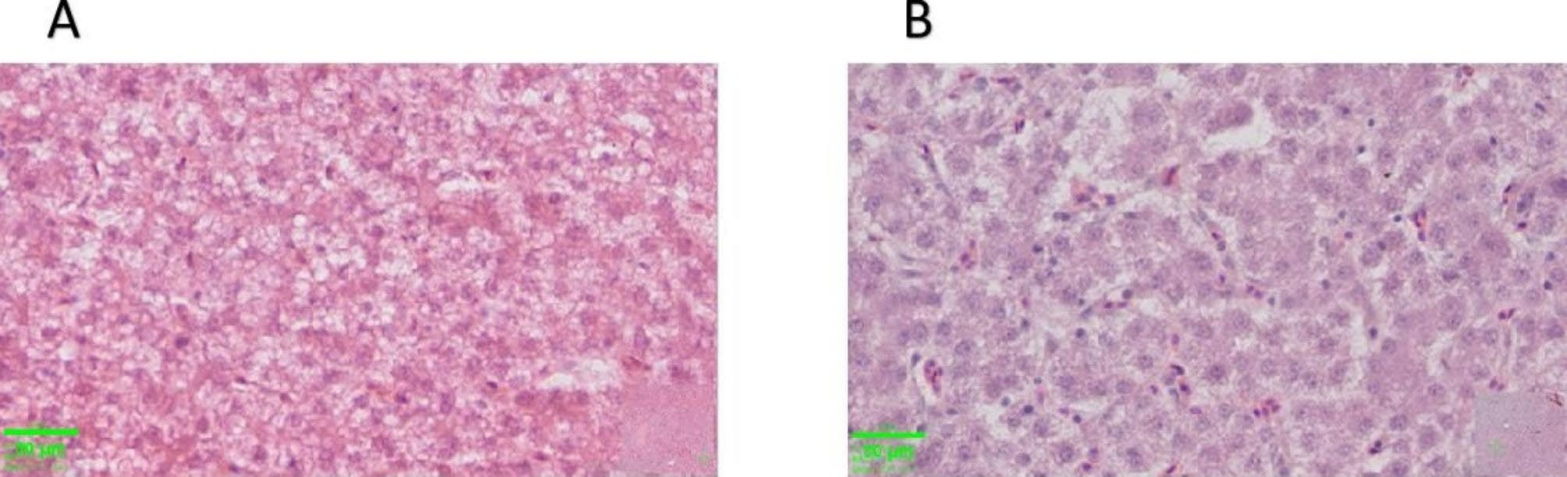
Effects of LGG on hepatic lipid in laying hens. Liver histology of CON group (**A**) and LGG group (**B**). Scale bar: 30 μm

The effects of LGG addition on laying hens’ serum and liver lipid metabolism indicators are shown in Table 4. The LDL (low-density lipoprotein) (*P* < 0.05) and FFA (free fatty acid) (*P* < 0.05) concentrations were markedly decreased by LGG supplementation in the serum. Nonetheless, the serum concentrations of TG and T-CHO were not significantly different between the two groups. In addition, compared to that in the CON group, the hepatic level of TG was decreased (*P* < 0.05) in the LGG group. Similar to the liver TG content, there was a visible reduction in liver lipid droplets after dietary LGG supplementation (Fig. 2).

**Table 4:** Effect of dietary LGG supplementation on serum lipid parameters of laying hens

Item	CON^1^	LGG^2^	Pooled SEM^3^	*P*-value
Serum (mmol/L)				
TG	21.8	18.0	1.96	0.190
T-CHO	3.98	3.46	0.300	0.240
LDL	1.76	1.11	0.210	0.0500
FFA	0.560	0.340	0.0500	0.0100
Liver (mmol/g)				
TG	0.440	0.330	0.0300	0.0200
T-CHO	0.0300	0.0300	0.0020	0.510

Abbreviations: TG = Triglyceride; T-CHO = Total- cholesterol; LDL = low-density lipoprotein; FFA = free fatty acids

^1^CON: basal diet

^2^LGG: basal diet supplemented with 8?1010 CFU/Kg LGG

^3^Data are expressed as the means and pooled standard error of the mean (Pooled SEM)

## Discussion

The laying rate of hens gradually declines in the late laying period, which accounts for approximately half of the laying period. The particular physiological characteristics of laying hens give rise to a series of health problems in the late laying period, including declines in nutrient absorption capacity and antibody performance and disorders of lipid metabolism. These changes in the physiological state weaken production performance and egg quality, causing significant economic impacts on farmers. This study showed that LGG added to the diet of hens in the late laying period significantly reduced the feed conversion rate, similar to a previous report showing that *Bifidobacterium spp.* and *Lactobacillus casei* significantly improved the feed conversion rate [28]. It is also noteworthy that the feed conversion rate, egg yield, egg weight, and egg quality were all greatly enhanced by adding *Enterococcus faecalis* (order Lactobacillales) to the diet [29]. The beneficial effects of probiotics on egg production may be due to their ability to boost intestinal health, reduce the stress response, and improve immune function [30–32].

Approximately 10–15% of eggs are broken during collection, storage, or transport due to eggshell quality problems [33]. Crucially, with older hens in the late laying period, egg weight increases, but eggshell weight does not, so the eggshell becomes thinner and its strength is decreased [34, 35]. Furthermore, in the late laying period, the weakened regeneration of endometrial cells, insufficient secretion of uterine fluid, and abnormal mineralization disrupt the uniformity of calcium deposition in eggshells, affecting their thickness and uniformity and reducing their strength [36]. This significant decrease in eggshell quality in the late laying period remains an important problem in breeding laying hens [37]. *Clostridium butyricum* added to the diet in the late laying period is reported to greatly enhance eggshell strength [38]. Late-period dietary probiotics had no effect on eggshell strength but significantly reduced the eggshell breakage rate and nonshell egg yield [39]. The present study found that LGG significantly increased eggshell thickness and eggshell strength but had no significant effect on Haugh units and yolk color in the late laying period.

The factors influencing eggshell strength are highly complex. The ultrastructure of the eggshell is a key factor determining its quality [40], and microscopic ultrastructural observation is helpful to better understand eggshell structure. While the benefits of probiotics on eggshell strength have been reported, their effect on the ultrastructure has rarely been studied. Eggshells have a highly ordered structure consisting of the inner and outer membranes, mammillary layer, palisade layer, vertical crystal layer, and cuticle. The effective thickness is the total thickness of the eggshell minus the effective mammillary thickness. In this study, LGG increased the effective layer thickness and density of the mammillary layer, reduced the width of the mastoid knot, and improved the surface cracking and pores of the eggshell. Generally, a higher density mammillary layer, smaller mastoid knot, and thicker palisade layer result in higher eggshell strength [41, 42]. An abnormally structured mammillary layer reduces its fracture resistance and can reduce the effective layer thickness or increase the porosity [41, 43], thus weakening the eggshell’s resistance to external forces. This study demonstrates that LGG can improve eggshell quality by altering its ultrastructure.

Serum biochemical indicators can be used to objectively assess animal health and metabolism status. Laying hens are prone to lipid metabolism disorders and various diseases during the late laying period, which follows the peak laying period during which a high metabolic output is required [44]. Our pilot study demonstrated the capability of probiotics to ameliorate lipid metabolism disorders in animals [25]. Probiotics play an important role in the regulation of lipid metabolism in poultry [45, 46]. It is particularly noteworthy that the probiotic *Clostridium butyricum* regulates the lipid metabolism of late-period laying hens by regulating the intestinal flora and the spectrum of bile acids [13] and that combining probiotics greatly improves serum biochemical indicators [47]. Furthermore, dietary *Lactobacillus acidophilus* significantly lowers the concentration of glycerol, total cholesterol, and low-density lipoprotein in the peripheral blood of laying hens [12]. This study found that LGG could effectively reduce the content of low-density lipoprotein and nonesterified fatty acids in serum and the content of triglycerides in liver tissue. It is important to control the lipid metabolism of late-period laying hens for optimum animal welfare and breeding purposes. Therefore, this strategy may also be an effective way for LGG to improve both production performance and egg quality.

## Conclusion

In conclusion, this study demonstrated that supplementation with 8 × 10^10^ CFU/kg LGG can improve hen performance and eggshell quality of laying hens during the late laying period. The positive effect of LGG on eggshell quality may be associated with improvement in the eggshell ultrastructure. In addition, our study provides evidence that dietary LGG also improves lipid metabolism indicators in laying hens. Therefore, dietary LGG supplementation could be recommended to positively impact the performance and health of laying hens.

## Methods

### Bacterial strain and culture conditions

The LGG strains were purchased from American Type Culture Collection (ATCC) (53,103) and cultured for 18 h in MRS broth medium at 5% CO_2_ and 37 °C.

Experimental Design.

The laying hens used in this experiment were self-bred and raised by the College of Animal Science and Technology, Jilin Agricultural Science and Technology University. Sixty healthy, non-antibiotic-fed Jinghong No. 1 laying hens (63 weeks old) were randomly divided into the control (CON) and LGG groups, with 5 laying hens in each replicate and 6 replicates in each group. The CON groups were fed a basal diet, while the LGG group was fed a diet containing 8 × 10^10^ CFU/kg LGG. The experimental period was 45 days total, with phases of 1–15 days, 16–30 days, and 31–45 days. Table 5 shows the nutritional level and composition of the basic diet.

**Table 5:** Dietary composition and nutrient levels of the experimental diets (as fed basis)

Ingredients	%	Nutrient content 3	Value
Corn	59.0	Metabolizable energy (MC/kg)	2.60
Soybean meal (43)	18.2	Crude protein (%)	15.5
Stone grain (10–20 mesh)	9.73	fibre(%)	2.94
DDGS	8.47	Calcium (%)	3.70
Rice bran meal (15.1%)	2.00	Total phosphorus (%)	0.470
Corn germ cake (16.7%)	0.790	Salt (%)	0.360
Rice bran oil	0.600	K (%)	0.620
Bone Calcium hydrogen phosphate	0.500	Na (%)	0.200
NaCl	0.250	Cl (%)	0.200
Methionine	0.120	Lysine (%)	0.750
Lysine sulfate(70%)	0.100	Methionine (%)	0.370
Mineral premix 1	0.100	DL-Methionine (%)	0.630
Choline chloride(50%)	0.100	Threonine (%)	0.580
Vitamin Premix 2	0.0300	Tryptophan (%)	0.160
Thermostable phytase(20,000)	0.0100	Isoleucine (%)	0.600
Total	100	Valine (%)	0.720

Abbreviations: DDGS = Distillers Dried Grains with Solubles

^1^Mineral premix provided the following per kg of diets: Cu,7,000 mg; Fe, 78,000 mg; Zn, 65,800 mg; Mn, 85,000 mg; I, 550 mg; Se, 300 mg

^2^Vitamin premix provided the following per kg of diets: VA, 4,000 IU; VD3, 1,500 IU; VK3, 15,000 mg; VE, 95,000 mg; VB1,10,000 mg; VB2, 30,000 mg; VB6, 15,000 mg; VB12, 120 mg; Nicotinamide, 150,000 mg; D biotin, 500 mg; Folic acid, 8,000 mg; D pantothenic acid, 45,000

^3^The values of metabolizable energy, available phosphorus, and amino acids are calculated, and others are measured values

### Growth conditions of laying hens and sample collection

Food and water were *ad libitum* accessible throughout the whole experimental period. Chickens were raised under artificial and natural lights for 16 h/d. The room temperature was monitored at 17–23 °C. Eggs were collected and weighed daily. At the end of the experiment, the chickens were fasted for 12 h prior to slaughter. Wing vein blood collection was performed. After stunning, cervical dislocation was performed.

### Production performance

The laying rates and feed conversion ratios were calculated. The laying rate was measured as the rate of egg production (e.g., broken and normal eggs) from each hen daily. The feed conversion ratio = feed consumption (kg)/total egg weight (kg).

### Egg quality

At the end of 45 days, 20 eggs were collected from the two groups to evaluate egg quality. The weight of each egg was recorded. An egg shape determinator (FHK, Japan) was used to determine the egg shape index. An eggshell strength tester (FHK, Japan) was employed to measure eggshell strength. An eggshell thickness gauge (FHK, Japan) was applied to measure eggshell thickness at different locations (middle, lower and upper end), and the mean value was calculated. The height of the albumen was measured at the three locations using an egg white height tester (FHK, Japan). After separation, the yolk color was determined using a colorimetric fan. Based on these data, the Haugh units were calculated. Haugh unit = 100 × log (AH + 7.57–1.7×EW^0.37^), where AH and EW are the albumen height (mm) and egg weight (g), respectively. Egg shape index = egg long diameter (longitudinal diameter)/egg short diameter (horizontal diameter).

### Eggshell ultrastructure

Three eggs from each group were randomly selected. The ultrastructure of an eggshell (0.5 ~ 1 cm^2^) was assessed using a scanning electron microscope. To facilitate membrane removal, eggshells were boiled in 2% NaOH for 10 min. Afterward, the shells were rinsed in water and dried for at least 24 h at room temperature. Thereafter, the eggshell samples were secured tightly on a conductive carbon film (double-sided adhesive) and analyzed using an ion sputter (Hitachi, Japan) for approximately 30 s. Subsequently, the eggshell cross-sections and inner and outer surfaces were observed using a scanning electron microscope (Hitachi, Japan). The effective thickness, mammillary thickness, and width of the mammillary knob were measured and averaged. The mammillary thickness was measured as the length from the top of the membrane to the bottom of the palisade. The average size of the mammillary knob was determined as follows: width = the length of the mammillary knob/the number of mammillary knobs. The percentages of effective thickness and mammillary thickness were calculated by measuring the ratio of the thickness of each layer to the total thickness. The cross-sectional images of each group were visualized under 200× magnification. For the inner and outer surface analysis, the images for each group were visualized under 100× magnification.

### Lipid metabolism parameters

The blood samples were stored at room temperature for 2 h, followed by centrifugation (2500 rpm, 30 min). The serum samples were stored at -70 °C until subsequent analyses. Nine samples from each group were randomly selected for low-density lipoprotein (LDL), total cholesterol (T-CHO), triglyceride (TG) and free fatty acid (FFA) measurement, which were measured by ELISA kits (Nanjing Jiancheng Institute of Bioengineering, China).

Then, nine liver samples were also randomly selected, and 0.1 g of liver tissue (1:9, w/v) was homogenized with 0.9% sodium chloride buffer using a SCIENTZ-48 L homogenizer (Ningbo Scientz Biotechnology, China). After centrifugation (4000×g, 15 min, 4 °C), the supernatant was subjected to measurement of the TG and T-CHO concentrations (Nanjing Jiancheng Institute of Bioengineering, China).

### Liver morphology analysis

The liver tissues were fixed in paraformaldehyde (10%) embedded in paraffin, and three randomly selected fixed liver tissues were embedded in paraffin and sectioned at a thickness of 5 mm. Subsequently, the slides were subjected to hematoxylin-eosin (H&E) staining. An Olympus BX43 microscope (Olympus Corp., Japan) was used to examine the stained sections.

### Statistical analysis

All values are shown as the mean and pooled SEM and were analyzed statistically by T test using GraphPad Prism 9. If the data did not conform to normality and lognormality tests, the Mann‒Whitney test was used. A *P* value < 0.05 was deemed statistically significant.

## Data Availability

The datasets used and/or analyzed during the current study are available from the corresponding author on reasonable request.

## Abbreviations

LDL: Low-density lipoprotein
T-CHO: Total cholesterol
TG: Triglycerides
FFA: Free fatty acids

## References

[1] Chen F, Zhang H, Du E, Jin F, Zheng C, Fan Q (2021). Effects of magnolol on egg production, egg quality, antioxidant capacity, and intestinal health of laying hens in the late phase of the laying cycle. Poult Sci.

[2] Guo Y, Zhao ZH, Pan ZY, An LL, Balasubramanian B, Liu WC (2020). New insights into the role of dietary marine-derived polysaccharides on productive performance, egg quality, antioxidant capacity, and jejunal morphology in late-phase laying hens. Poult Sci.

[3] Zhang Y, Ma W, Zhang Z, Liu F, Wang J, Yin Y (2019). Effects of Enterococcus faecalis on egg production, egg quality and caecal microbiota of hens during the late laying period. Arch Anim Nutr.

[4] Mi J, Chen X, Liao X (2019). Screening of single or combined administration of 9 probiotics to reduce ammonia emissions from laying hens. Poult Sci.

[5] Agazzi A. The beneficial role of Probiotics in Monogastric Animal Nutrition and Health. J Dairy Veterinary Anim Res. 2015;2(4).

[6] Xiang Q, Wang C, Zhang H, Lai W, Wei H, Peng J (2019). Effects of different probiotics on laying performance, Egg Quality, oxidative status, and Gut Health in laying hens. Animals(Basel).

[7] Park JW, Jeong JS, Lee SI, Kim IH (2016). Effect of dietary supplementation with a probiotic (Enterococcus faecium) on production performance, excreta microflora, ammonia emission, and nutrient utilization in ISA brown laying hens. PoultSci.

[8] Zhou Y, Li S, Pang Q, Miao Z (2020). Bacillus amyloliquefaciens BLCC1-0238 can effectively improve laying performance and Egg Quality Via enhancing immunity and regulating Reproductive Hormones of laying hens. ProbioticsAntimicrobProteins.

[9] Liu X, Peng C, Qu X, Guo S, Chen JF, He C (2019). Effects of Bacillus subtilis C-3102 on production, hatching performance, egg quality, serum antioxidant capacity and immune response of laying breeders. J Anim Physiol Anim Nutr.

[10] Siadati SA, Salehi Jouzani YEG, Shayegh J. Evaluation of the Probiotic Potential of some Native Lactobacillus Strains on the Laying Performance and Egg Quality Parameters of Japanese Quails. Iran J Appl Anim Sci. 2018;8(4):703–12.

[11] Tang SGH, Sieo CC, Ramasamy K, Saad WZ, Wong HK, Ho YW (2017). Performance, biochemical and haematological responses, and relative organ weights of laying hens fed diets supplemented with prebiotic, probiotic and synbiotic. BMC Vet Res.

[12] Alaqil AAA, El-Beltagi AO, El-Atty HS, A.Mehaisen HK, Moustafa GMK. Dietary supplementation of probiotic Lactobacillus acidophilus modulates cholesterol levels, Immune Response, and productive performance of laying hens. Anim (Basel). 2020;10(9). 10.3390/ani10091588 PMC7552304 32899938

[13] Wang WW, Wang J, Zhang HJ, Wu SG, Qi GH (2020). Supplemental Clostridium butyricum modulates lipid metabolism through shaping gut microbiota and bile Acid Profile of aged laying hens. FrontMicrobiol.

[14] Wei F, Yang X, Zhang M, Xu C, Hu Y, Liu D (2022). Akkermansia muciniphila enhances Egg Quality and the lipid Profile of Egg yolk by improving lipid metabolism. FrontMicrobiol.

[15] Chen JF, Zhuang Y, Jin SB, Zhang SL, Yang WW (2021). Probiotic Lactobacillus rhamnosus GG (LGG) restores intestinal dysbacteriosis to alleviate upregulated inflammatory cytokines triggered by femoral diaphyseal fracture in adolescent rodent model. Eur Rev Med Pharmacol Sci.

[16] Shi CW, Cheng MY, Yang X, Lu YY, Yin HD, Zeng Y (2020). Probiotic Lactobacillus rhamnosus GG promotes mouse gut microbiota diversity and T cell differentiation. FrontMicrobiol.

[17] Guo M, Zhang C, Zhang C, Zhang X, Wu Y (2021). Lacticaseibacillus rhamnosus reduces the pathogenicity of Escherichia coli in chickens. Front Microbiol.

[18] He X, Zeng Q, Puthiyakunnon S, Zeng Z, Yang W, Qiu J (2017). Lactobacillus rhamnosus GG supernatant enhance neonatal resistance to systemic Escherichia coli K1 infection by accelerating development of intestinal defense. Sci Rep.

[19] Chen L, Li S, Peng C, Gui Q, Li J, Xu Z et al. Lactobacillus rhamnosus GG promotes recovery of the Colon barrier in septic mice through accelerating ISCs regeneration. Nutrients. 2023;15(3). 10.3390/nu15030672 PMC9921111 36771378

[20] Wang Y, Gong L, Wu YP, Cui ZW, Wang YQ, Huang Y (2019). Oral administration of Lactobacillus rhamnosus GG to newborn piglets augments gut barrier function in pre-weaning piglets. J Zhejiang Univ Sci B.

[21] Bai Y, Ma K, Li J, Li J, Bi C, Shan A (2021). Deoxynivalenol exposure induces liver damage in mice: inflammation and immune responses, oxidative stress, and protective effects of Lactobacillus rhamnosus GG. Food Chem Toxicol.

[22] Voo PY, Wu CT, Sun HL, Ko JL, Lue KH (2022). Effect of combination treatment with Lactobacillus rhamnosus and corticosteroid in reducing airway inflammation in a mouse asthma model. J Microbiol Immunol Infect.

[23] Kim SW, Park KY, Kim B, Kim E, Hyun CK (2013). Lactobacillus rhamnosus GG improves insulin sensitivity and reduces adiposity in high-fat diet-fed mice through enhancement of adiponectin production. Biochem Biophys Res Commun.

[24] Schneider AC, Machado AB, de Assis AM, Hermes DM, Schaefer PG, Guizzo R (2014). Effects of Lactobacillus rhamnosus GG on hepatic and serum lipid profiles in zebrafish exposed to ethanol. Zebrafish.

[25] Zhao C, Liu L, Liu Q, Li F, Zhang L, Zhu F (2019). Fibroblast growth factor 21 is required for the therapeutic effects of Lactobacillus rhamnosus GG against fructose-induced fatty liver in mice. Mol Metab.

[26] Zhao H, Zhao C, Dong Y, Zhang M, Wang Y, Li F (2015). Inhibition of miR122a by Lactobacillus rhamnosus GG culture supernatant increases intestinal occludin expression and protects mice from alcoholic liver disease. Toxicol Lett.

[27] Zhang M, Wang C, Wang C, Zhao H, Zhao C, Chen Y (2015). Enhanced AMPK phosphorylation contributes to the beneficial effects of Lactobacillus rhamnosus GG supernatant on chronic-alcohol-induced fatty liver disease. J Nutr Biochem.

[28] Lokapirnasari WP, Pribadi TB, Arif AA, Soeharsono S, Hidanah S, Harijani N (2019). Potency of probiotics Bifidobacterium spp. and Lactobacillus casei to improve growth performance and business analysis in organic laying hens. Vet World.

[29] Mikulski D, Jankowski J, Mikulska M, Demey V (2020). Effects of dietary probiotic (Pediococcus acidilactici) supplementation on productive performance, egg quality, and body composition in laying hens fed diets varying in energy density. Poult Sci.

[30] Deng W, Tong XFDJM, Zhang Q (2012). The probiotic Bacillus licheniformis ameliorates heat stress-induced impairment of egg production, gut morphology, and intestinal mucosal immunity in laying hens. Poult Sci.

[31] Lei K, Li YL, Yu DY, Rajput IR, Li WF (2013). Influence of dietary inclusion of Bacillus licheniformis on laying performance, egg quality, antioxidant enzyme activities, and intestinal barrier function of laying hens. Poult Sci.

[32] Forte C, Moscati L, Acuti G, Mugnai C, Franciosini MP, Costarelli S (2016). Effects of dietary Lactobacillus acidophilus and Bacillus subtilis on laying performance, egg quality, blood biochemistry and immune response of organic laying hens. J Anim Physiol Anim Nutr (Berl).

[33] Stefanello C, Santos TC, Murakami AE, Martins EN, Carneiro TC (2014). Productive performance, eggshell quality, and eggshell ultrastructure of laying hens fed diets supplemented with organic trace minerals. Poult Sci.

[34] Molnar A, Maertens L, Ampe B, Buyse J, Kempen I, Zoons J (2016). Changes in egg quality traits during the last phase of production: is there potential for an extended laying cycle?. Br Poult Sci.

[35] Roberts JR, Chousalkar K, Samiullah. Egg quality and age of laying hens: implications for product safety. Anim Prod Sci. 2013;53(12).

[36] Huntley DMHDP. Ultrastructure of shell gland tissue from hens producing good and poor eggshells Poultry Science. 1978:57:1365–8.

[37] Fathi MM, Galal A, Ali UM, Abou-Emera OK (2019). Physical and mechanical properties of eggshell as affected by chicken breed and flock age. Br Poult Sci.

[38] Zhan HQ, Dong XY, Li LL, Zheng YX, Gong YJ, Zou XT (2019). Effects of dietary supplementation with Clostridium butyricum on laying performance, egg quality, serum parameters, and cecal microflora of laying hens in the late phase of production. Poult Sci.

[39] Yan FF, Murugesan GR, Cheng HW (2019). Effects of probiotic supplementation on performance traits, bone mineralization, cecal microbial composition, cytokines and corticosterone in laying hens. Animal.

[40] Ketta M, Tůmová E (2016). Eggshell structure, measurements, and quality-affecting factors in laying hens: a review. Czech J Anim Sci.

[41] Radwan ML. Eggshell quality: a comparison between Fayoumi, Gimieizah and Brown Hy-Line strains for mechanical properties and ultrastructure of their eggshells. Anim Prod Sci. 2016;56(5).

[42] Li LL, Zhang NN, Gong YJ, Zhou MY, Zhan HQ, Zou XT (2018). Effects of dietary Mn-methionine supplementation on the egg quality of laying hens. Poult Sci.

[43] Dunn IC, Rodrı´guez-Navarro AB, Mcdade K, Schmutz M, Preisinger R, Waddington D (2012). Genetic variation in eggshell crystal size and orientation is large and these traits are correlated with shell thickness and are associated with eggshell matrix protein markers. Anim Genet.

[44] Liu XT, Lin X, Mi YL, Zeng WD, Zhang CQ (2018). Age-related changes of yolk precursor formation in the liver of laying hens. J Zhejiang Univ Sci B.

[45] Saleh AA, Eid YZ, Ebeid TA, Ohtsuka A, Hioki K, Yamamoto M (2012). The modification of the muscle fatty acid profile by dietary supplementation with aspergillus awamori in broiler chickens. Br J Nutr.

[46] Baghban-Kanani P, Hosseintabar-Ghasemabad B, Azimi-Youvalari S, Seidavi A, Ragni M, Laudadio V (2019). Effects of using Artemisia annua Leaves, Probiotic Blend, and Organic acids on performance, Egg Quality, Blood Biochemistry, and antioxidant status of laying hens. J Poult Sci.

[47] Naseem S, King AJ (2020). Effects of Multi-Species Lactobacillus and sunflower seed meal on Nitrogen-Containing Compounds in laying hens’ manure and Biological Components in Blood serum. J Appl Poult Res.

